# Intubation duration after general anesthesia for thrombectomy in acute ischemic stroke: a cohort study

**DOI:** 10.3389/fneur.2026.1937683

**Published:** 2026-08-27

**Authors:** Jingjing Fan, Guoyan Liu, Yun Du, Zhiguo Hao, Jinfeng Yang, Shuqin Zhan, Dacheng Liu

**Affiliations:** Department of Neurology, The Second Affiliated Hospital of Xi’an Jiaotong University, Xi’an, China

**Keywords:** acute ischemic stroke, endovascular thrombectomy, general anesthesia, intubation duration, prognosis

## Abstract

**Objective:**

General anesthesia (GA) is commonly used for acute ischemic stroke (AIS) patients undergoing endovascular thrombectomy. However, the timing of extubation post thrombectomy remains unclear. We aimed to investigate the association of intubation duration and outcomes in these patients.

**Methods:**

AIS patients receiving acute endovascular thrombectomy under GA were consecutively enrolled. Patients with intubation duration ≥ 96 h and discharged/died with intubation within 96 h were excluded. Adjusted odds ratio (aOR) of intubation duration over the outcomes were obtained. The primary outcome was modified Rankin Scale (mRS) 3–6 at 90 days. Secondary outcomes included pneumonia during hospitalization, 90-day mortality and intracranial hemorrhagic transformation.

**Results:**

133 patients were enrolled in final analysis. 69 patients (51.9%) had mRS 3–6 at 90 days. Pneumonia occurred in 74 patients (55.6%). 14 (10.5%) patients died within 90 days follow-up. 31(23.3%) patients experienced intracranial hemorrhagic transformation. In multivariable analysis, longer intubation duration was associated with higher risk of 90-d mRS 3–6 (aOR 1.19 per 6-h increment, 95% CI 1.04–1.35, *p* = 0.01) and pneumonia (aOR 1.28 per 6-h increment, 95% CI 1.12–1.46, *p* < 0.001).

**Conclusion:**

Prolonged intubation duration post-thrombectomy in AIS under GA is associated with increased risk of poor outcome and pneumonia. Intubation duration may serve as a prognostic marker in this setting. But prospective studies are needed to determine whether early extubation improves outcomes.

## 1 Introduction

Endovascular thrombectomy is the first-line treatment for patients with acute ischemic stroke (AIS) due to large vessel occlusion who present within the therapeutic time window. Conscious sedation (CS) and general anesthesia (GA) are the two main anesthetic modalities for thrombectomy (1). Recent randomized controlled trials have demonstrated no significant differences in recanalization rates or clinical outcomes between the two anesthetic approaches (2–5). Compared with CS, GA may prolong the time from hospital admission to arterial puncture; however, it offers better patient cooperation and therefore remains a commonly used anesthetic modality in clinical practice (6). Nevertheless, for patients undergoing thrombectomy under GA with endotracheal intubation, the optimal timing of extubation after the procedure currently lacks clear evidence-based guidance.

Two previous observational studies suggested that prolonged intubation duration after thrombectomy was associated with poor outcomes (7, 8); however, both studies were limited to patients with anterior circulation stroke treated within a narrow time window. A recently published Early vs. Delayed Extubation After Thrombectomy for Acute Ischemic Stroke (ENESTROKE) trial (9), which enrolled patients with complete reperfusion under GA within 24 h of onset, found that extubation within 6 h after thrombectomy did not significantly improve outcomes compared with extubation within 12 h. This trial suggests that very early extubation may not be beneficial. Theoretically, delayed extubation may increase the risk of post-stroke pneumonia, and prolonged use of sedatives and analgesics may affect hemodynamics, thereby influencing clinical outcomes (10). Conversely, premature extubation may induce blood pressure fluctuations and increase the risk of hyperperfusion syndrome, particularly in patients with impaired cerebral autoregulation after reperfusion (11). In addition, it may lead to higher reintubation rates in selected patients (e.g., those with dysphagia or impaired consciousness) (12). To investigate the association between intubation duration and outcomes after thrombectomy under GA, we conducted the present cohort study.

## 2 Methods

### 2.1 Study design and participants

This was a single center cohort study. Eligible participants met the following inclusion criteria: (1) acute ischemic stroke within 24 h of onset; (2) large vessel occlusion, including the basilar artery, V4 segment of the vertebral artery, internal carotid artery, M1/M2 segment of the middle cerebral artery, or A1 segment of the anterior cerebral artery; (3) endovascular thrombectomy performed under GA; (4) duration of mechanical ventilation ≤96 h; and (5) completion of 90-day follow-up. Patients who died while intubated within 96 h or were discharged with the endotracheal tube in place within 96 h for any reason were excluded from the final analysis.

### 2.2 Anesthesia selection and perioperative management

Both GA and CS were routinely used for thrombectomy at our center. The choice of anesthesia was determined at the discretion of the operating physician, and no standardized institutional protocol was applied. However, GA was favored in cases of significant agitation precluding cooperation, impaired airway protection with high aspiration risk, or anticipated complex procedures requiring prolonged intervention.

All patients were admitted to the neurointensive care unit after thrombectomy, and peri-operative management followed standard clinical practice and guideline. Routine sedation and analgesia were provided to all patients after thrombectomy under GA, with midazolam and remifentanil as the primary agents. Prior to extubation, all sedatives were withdrawn, with the exception of a low-dose remifentanil infusion, which could be maintained for postoperative analgesia.

### 2.3 Extubation strategy

The decision to extubate was made individually by the attending physician based on the patient’s clinical status and standard institutional practice. Before extubation, all patients routinely underwent head Computed Tomography to rule out contraindications including symptomatic intracranial hemorrhage and malignant cerebral edema. Following sedation withdrawal, patients were considered eligible for extubation if they met the following criteria: ability to follow commands, hemodynamic stability, presence of cough reflex, adequate spontaneous breathing without respiratory distress, and successful completion of a spontaneous breathing trial (SBT) (13). The intubation duration was recorded for each patient. An intubation duration exceeding 24 h was defined as delayed extubation, and the reasons for delayed extubation were routinely documented in the medical records.

### 2.4 Data collection

Baseline clinical data were collected for all patients, including age, sex, previous medical history, stroke severity assessed by the National Institutes of Health Stroke Scale (NIHSS), level of consciousness assessed by Glasgow Coma Scale (GCS), decreased or absent cough reflex, admission blood pressure, and baseline laboratory findings (blood glucose, renal function, complete blood count, etc.). The first cranial computed tomography (CT) or diffusion weighted magnetic resonance imaging was used to evaluate the Alberta Stroke Program Early CT Score (ASPECTS) as a measure of infarct volume (14). Stroke etiology was documented according to the Trial of Org 10,172 in Acute Stroke Treatment (TOAST) classification (15). Procedure-related information was also recorded: culprit vessel, post-procedural reperfusion grade according to the modified Thrombolysis in Cerebral Infarction (mTICI) scale (16), time from onset to arterial puncture, time from puncture to recanalization, and collateral status graded by the American Society of Interventional and Therapeutic Neuroradiology/Society of Interventional Radiology grading system (ASITN/SIR) (17).

### 2.5 Outcomes

The primary outcome was poor outcome at 90 days, defined as functional dependence or death (modified Rankin Scale [mRS] score 3–6). Secondary outcomes included 90-day mortality, hemorrhagic transformation within 48 h after the procedure, and pneumonia. Hemorrhagic transformation was classified according to the ECASS III (European Cooperative Acute Stroke Study III) criteria (18) into four types: hemorrhagic infarction 1, hemorrhagic infarction 2, parenchymal hemorrhage 1, parenchymal hemorrhage 2. Pneumonia was identified based on International Classification of Diseases, Tenth Revision (ICD-10) diagnosis codes obtained from the medical record face sheet.

### 2.6 Statistical analysis

Baseline characteristics were compared between the favorable and unfavorable outcome groups. Categorical variables were analyzed using the chi-square test or Fisher‘s exact test (when expected frequencies were <5), and continuous variables using Student’s t test or Mann–Whitney U test. Additionally, baseline comparisons were performed between the GA and CS groups, and between the included and excluded populations in GA group. Univariate and multivariable logistic regression analyses were used to assess the association between intubation duration (per 6 h increase) and outcomes, adjusting for age, sex, baseline NIHSS score, GCS score, cough reflex, ASPECTS score, renal function, intravenous thrombolysis, and atrial fibrillation. Results are presented as adjusted odds ratios (aOR) with 95% confidence intervals (CIs). The predicted probability of poor outcome was calculated based on the fitted logistic model. To test the linearity assumption, restricted cubic spline (RCS) analysis with 4 knots was performed, and nonlinearity was assessed by the Wald chi-square test. As a sensitivity analysis, intubation duration was categorized into three clinically predefined groups (≤24 h, 24-48 h, and >48 h) using the same models to explore threshold effects. All supplementary analyses were considered supportive to the primary continuous variable model. Exploratory receiver operating characteristic (ROC) curves were constructed to evaluate the predictive performance of intubation duration for poor outcome and pneumonia, and the area under the curve (AUC) was calculated. The optimal cut-off values were determined, and the corresponding sensitivity, specificity, positive predictive value, negative predictive value, and Youden index were reported. All statistical analyses were performed using R software (version 4.5.2). A two-sided *p*-value < 0.05 was considered statistically significant.

## 3 Results

### 3.1 Patients characteristics

**Figure 1:**
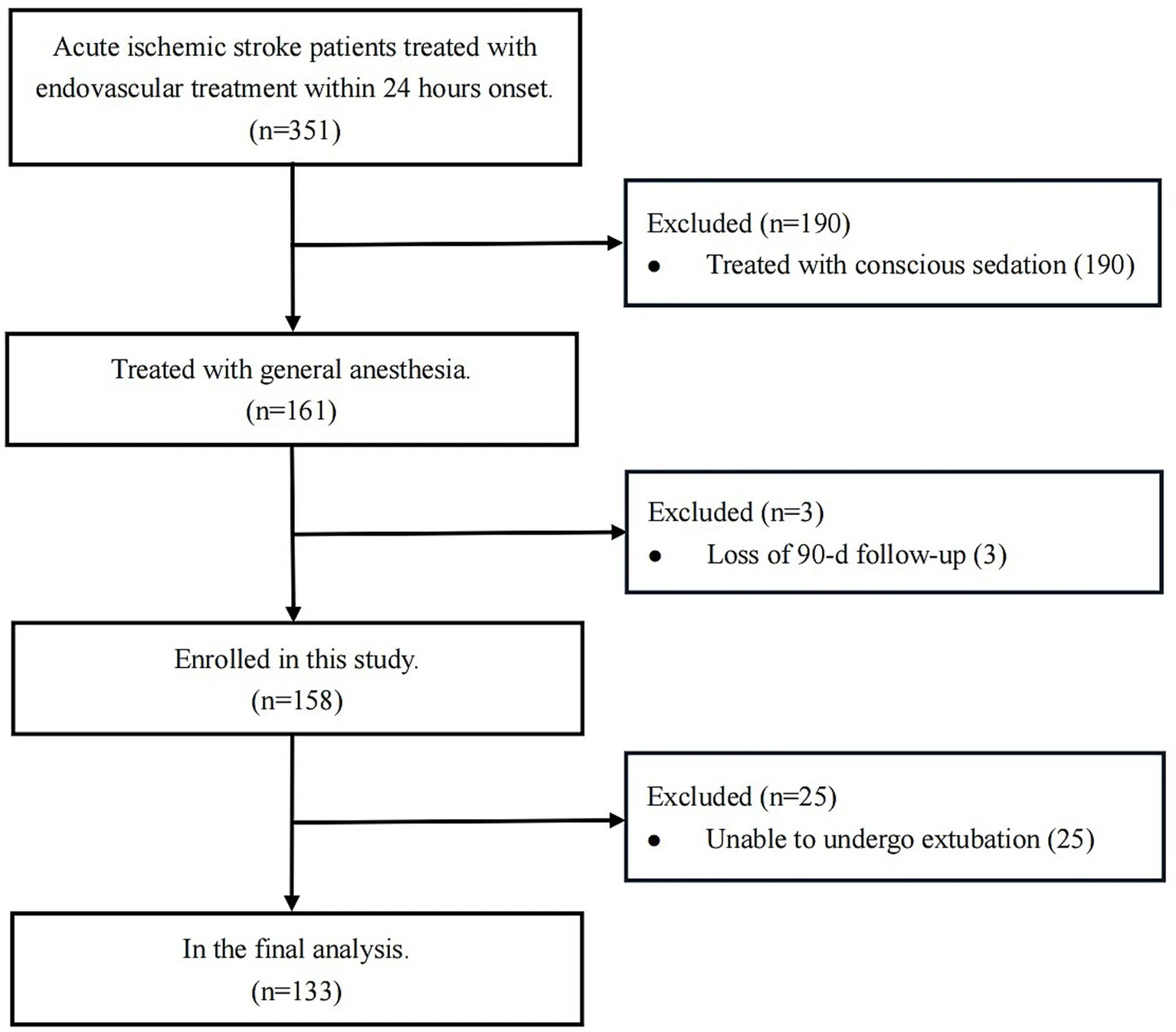
Flow-chart.

From January 2018 to September 2024, a total of 351 patients with AIS underwent emergency endovascular thrombectomy. Among them, 190 received CS and 161 received GA. Of the 161 GA patients, 3 were lost to follow-up, and 25 could not be extubated within 96 h post-thrombectomy for various reasons. Ultimately, 133 patients were included in the final analysis (Figure 1).

Baseline characteristics of patients with good (90-d mRS 0–2) versus poor (90-d mRS 3–6) outcome are shown in Table 1, the poor outcome group had a higher proportion of atrial fibrillation, higher proportion of decreased or absent cough reflex, higher baseline NIHSS score, lower GCS score, lower ASPECTS score, higher proportion of cardioembolic stroke, lower estimated glomerular filtration rate (eGFR), and a lower rate of intravenous thrombolysis (all *p* value<0.05). In addition, we compared baseline characteristics between CS and GA group (Supplementary Table S1), as well as between the 25 excluded patients who could not be extubated within 96 h under GA and the study cohort (Supplementary Table S2).

**Table 1:** Baseline characteristics between good and poor functional outcome.

Characteristics	90-d mRS 0–2 (*N* = 64)	d mRS 3–6 (*N* = 69)	*p* value
Male, *n* (%)	48 (75.0)	43 (62.3)	0.11
Age, mean (SD)	59.0 (12.4)	66.7 (10.3)	<0.001
Medical history, *n* (%)			
Hypertension	44 (68.8)	51 (73.9)	0.51
Diabetes	18 (28.1)	24 (34.8)	0.40
Atrial fibrillation	4 (6.3)	20 (29.0)	<0.001
Stroke	9 (14.1)	11 (15.9)	0.76
Coronary heart disease	8 (12.5)	12 (17.4)	0.43
Smoking	25 (39.1)	30 (43.5)	0.60
Baseline NIHSS, median (IQR)	9 (5–14)	15 (11–19)	<0.001
Baseline GCS, median (IQR)	14 (11–15)	12 (9–13)	<0.001
Baseline ASPECTS, median (IQR)	7 (5–8)	6 (5–7)	0.05
Baseline SBP, mmHg, mean (SD)	149.7 (23.1)	150.5 (19.1)	0.83
Baseline DBP, mmHg, mean (SD)	85.5 (14.3)	86.9 (13.8)	0.58
Cardiogenic embolism, *n* (%)	7 (10.9)	21 (30.4)	0.01
Decreased or absent cough reflex, *n* (%)	23 (35.9%)	50 (72.5%)	<0.001
Baseline laboratory test, mean (SD)			
Estimated glomerular filtration rate, mL/min per 1.73 m^2^	104.8 (19.5)	92.7 (22.1)	0.001
Blood glucose, mmol/l	7.2 (3.4)	7.2 (2.7)	0.98
White blood count, 10^9^/L	8.9 (3.5)	9.6 (3.6)	0.30
Vertebrobasilar artery occlusion, *n* (%)	19 (29.7)	22 (31.9)	0.66
Intravenous thrombolysis, *n* (%)	22 (34.4)	9 (13.0)	0.004
ASITN/SIR scale 3–4, *n* (%)	18 (28.2)	11 (15.9)	0.39
Onset to recanalization time, min, median (IQR)	614 (423–917)	605 (435–844)	0.83
mTICI scale 2b-3, *n* (%)	61 (95.3)	62 (89.9)	0.63
Off-hours, *n* (%)	18 (28.1)	23 (33.3)	0.52
Hemorrhagic transformation, *n* (%)			0.03
Hemorrhagic infarction 1	5 (7.8)	4 (5.8)	
Hemorrhagic infarction 2	4 (6.3)	4 (5.8)	
Parenchymal hemorrhage 1	0 (0)	6 (8.7)	
Parenchymal hemorrhage 2	1 (1.6)	7 (10.1)	
Pneumonia, *n* (%)	23 (35.9)	51 (73.9)	<0.001

mRS, modified Rankin Scale; SD, standard deviation; NIHSS, National Institutes of Health Stroke Scale score; IQR, interquartile ranges; GCS, Glasgow Coma Scale; ASPECTS, Alberta Stroke Program Early CT Score; SBP, systolic blood pressure; DBP, diastolic blood pressure; ASITN/SIR, American Society of Interventional and Therapeutic Neuroradiology/Society of Interventional Radiology; mTICI, modified Thrombolysis in Cerebral Infarction.

A total of 58 patients (43.6%) had an intubation duration >24 h and were classified as having delayed extubation. The causes included hemodynamic instability (*n* = 3), delayed emergence from anesthesia (*n* = 6), failed SBT due to severe hypoxemia (*n* = 4), and neurological causes (*n* = 45), such as severe impaired consciousness, massive infarction, and symptomatic hemorrhagic transformation, among others. In addition, three patients required reintubation after extubation: one due to laryngeal edema and two due to severe hypoxemia secondary to pulmonary infection.

### 3.2 Intubation duration and functional outcomes

69 patients (51.9%) had mRS 3–6 at 90 days. Pneumonia occurred in 74 patients (55.6%). 14 (10.5%) patients died within 90 days follow-up. 31 (23.3%) patients experienced intracranial hemorrhagic transformation.

In multivariable logistic regression models, after adjusting for age, sex, baseline NIHSS score, baseline GCS, baseline ASPECTS score, cough reflex, renal function, intravenous thrombolysis, and atrial fibrillation, longer intubation duration was associated with higher risk of 90-d mRS 3–6 (aOR 1.19 per 6-h increment, 95% CI 1.04–1.35, *p* = 0.01) and pneumonia (aOR 1.28 per 6-h increment, 95% CI 1.12–1.46, *p* < 0.001), but not with 90-d mortality or hemorrhagic transformation (Table 2).

**Table 2:** Effect of intubation duration on outcomes.

Outcomes	Unadjusted OR (95% CI)	*p* value	aOR (95% CI)*	*p* value
90-d mRS 3–6	1.23 (1.11–1.37)	<0.001	1.19 (1.04–1.35)	0.01
90-d mortality	1.13 (1.01–1.26)	0.02	1.01 (0.85–1.17)	0.96
Hemorrhagic transformation	0.93 (0.85–1.01)	0.08	0.93 (0.84–1.03)	0.17
Pneumonia	1.28 (1.14–1.45)	<0.001	1.28 (1.12–1.46)	<0.001

Odds ratio per 6 h intubation duration increment for outcomes. OR, odd ratio; aOR, adjusted OR; CI, confidence interval; mRS, modified Rankin Scale.

*Adjusting for age, sex, baseline National Institutes of Health Stroke Scale score, baseline Glasgow Coma Scale, baseline Alberta Stroke Program Early CT Score, cough reflex, baseline estimated glomerular filtration rate, intravenous thrombolysis and atrial fibrillation.

In further sensitivity analysis, compared with intubation duration ≤24 h, patients with intubation duration >48 h and higher risk of 90-d mRS 3–6 (aOR 2.81, 95% CI 1.15–6.87, *p* = 0.02) and pneumonia (aOR 3.10, 95% CI 1.09–8.80, *p* = 0.03), whereas the 24-48 h group did not show a significant association (Supplementary Table S3).

**Figure 2:**
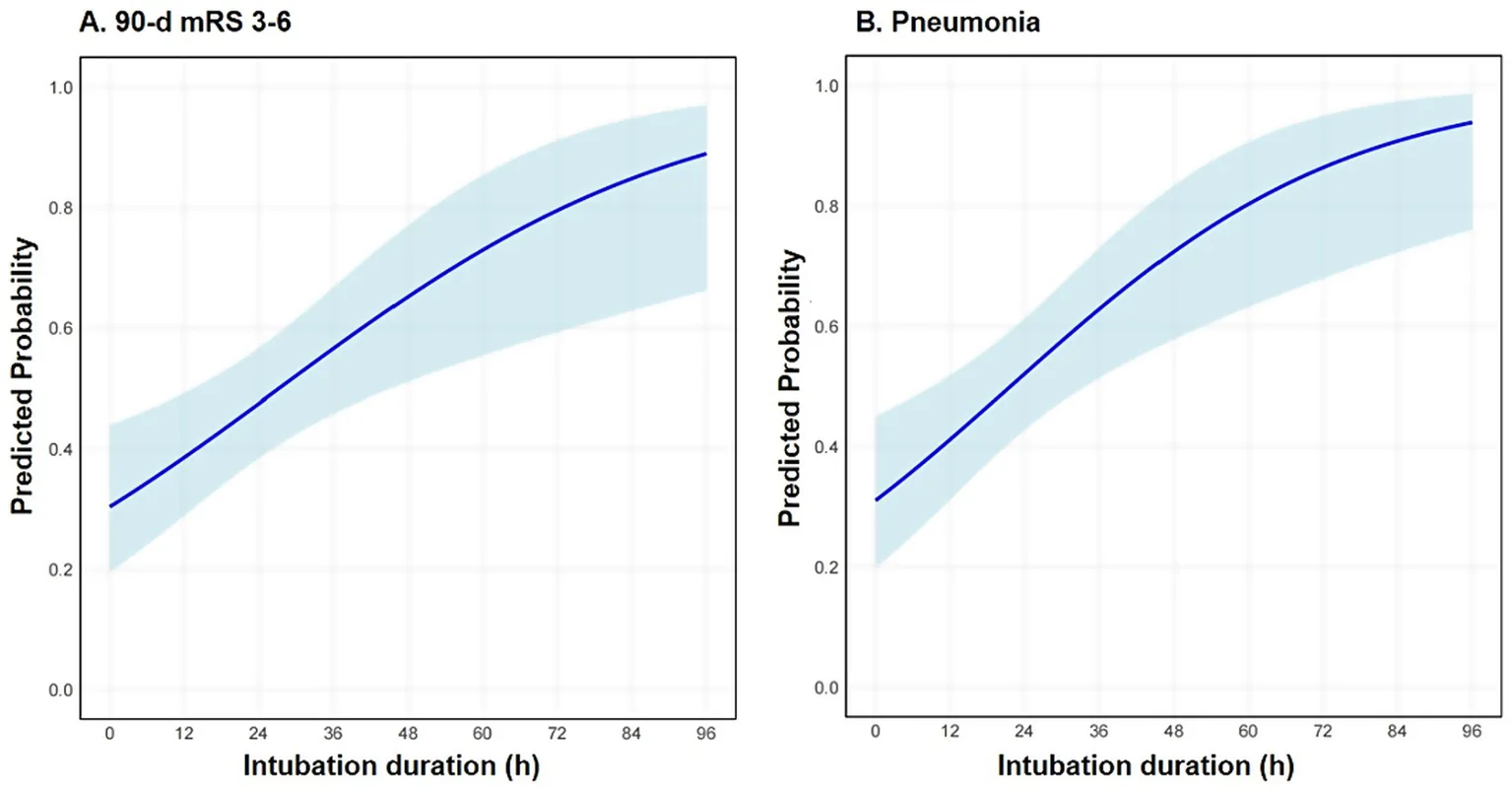
Relationship between intubation duration after endovascular treatment and outcomes. **(A)** The relationship between intubation duration after endovascular treatment and 90-d mRS 3–6. **(B)** The relationship between intubation duration after endovascular treatment and pneumonia during hospitalization. mRS, modified Rankin Scale.

The predicted probability of intubation duration for 90-d mRS 3–6 and pneumonia were displayed in Figure 2. Restricted cubic spline (RCS) analysis revealed no significant nonlinearity for either outcome (P for nonlinearity = 0.65 for mRS 3–6 and 0.33 for pneumonia).

### 3.3 Predictive value of intubation duration for functional outcomes

ROC curves were constructed to assess the predictive ability of intubation duration for poor outcome and pneumonia (Supplementary Figure S1). The AUC for predicting poor outcome was 0.71(95% CI 0.62–0.79), with an exploratory cutoff of 38 h, a Youden index of 0.35, sensitivity 0.46, specificity 0.89, positive predictive value 0.82, and negative predictive value 0.61. For predicting pneumonia, the AUC was 0.72 (95% CI 0.63–0.81), with an exploratory cutoff of 23.5 h, a Youden index of 0.41, sensitivity 0.65, specificity 0.76, positive predictive value 0.77, and negative predictive value 0.63 (Table 3).

**Table 3:** Predictive value of intubation duration for primary outcome and pneumonia.

Parameters	90-d mRS 3–6	Pneumonia
AUC (95% CI)	0.71 (0.62–0.79)	0.72 (0.63–0.81)
Cut-off value, h	38.0	23.5
Youden index	0.35	0.41
Sensitivity	0.46	0.65
Specificity	0.89	0.76
Positive predicative value	0.82	0.77
Negative predicative value	0.61	0.63

mRS, modified Rankin Scale; AUC, area under curve; CI, confidence interval.

## 4 Discussion

In this study, prolonged intubation duration after endovascular thrombectomy under GA in patients with large vessel occlusion was significantly associated with poor 90-day outcome and pneumonia in a time-dependent manner, even after adjustment for potential confounders.

Consistent with previous studies (7, 8), prolonged intubation duration was associated with an increased risk of poor outcome. However, unlike prior studies, our cohort included approximately 30% of patients with posterior circulation stroke, whereas previous reports mainly focused on anterior circulation strokes. Moreover, we excluded patients requiring mechanical ventilation longer than 96 h and those who died or were discharged with the tube in place within 96 h (all had poor outcomes, 100%, Supplementary Table S2). Because previous studies have clearly demonstrated that prolonged ventilation beyond 96 h is associated with poor outcomes (11, 19). This makes our findings more applicable to patients in whom extubation decisions can be actively made rather than those with extremely severe neurological damage precluding extubation. The exploratory cutoff values derived from ROC analysis are presented in Table 3 and Supplementary Figure S1; these findings should be interpreted with caution and require external validation.

In our cohort, patients with longer intubation duration had higher baseline NIHSS scores, lower GCS score, lower ASPECTS scores, a higher proportion of cardioembolic stroke, decreased or absent cough reflex, and worse renal function, indicating that these patients were more severely ill at baseline and less likely to be extubated early. Nevertheless, after multivariable adjustment for these confounders, each 6-h increase in intubaion duration remained associated with increased risk of poor outcome and pneumonia, suggesting that the association was not fully attributable to baseline disease severity alone.

Non-medical factors may also influence intubation duration. Previous studies have reported that procedures performed during off-hours (e.g., weekends or holidays) may increase the risk of poor functional outcome (20, 21). Extubation might be delayed due to reduced staffing in off-hours (7). However, in our study, we compared patients treated on holidays versus non-holidays and found no significant differences in outcomes or intubation duration groups.

The present study also found that the risk of pneumonia increased progressively with longer intubation duration, which is consistent with previous study (7). As intubation duration was further prolonged, the risk of pneumonia increased substantially. Once pneumonia occurred, it could exacerbate ischemic brain injury through systemic inflammatory responses, hypoxemia, and other mechanisms, thereby leading to poor outcome (22–24). Given the strong association between intubation duration and pneumonia observed in our cohort, enhanced pneumonia prevention strategies may be beneficial in patients with prolonged intubation, though this hypothesis requires prospective validation (25).

The incidence of pneumonia in our study was higher than that reported in previous studies (7, 8, 24), which may be attributable to differences in the definition of pneumonia. Specifically, we did not strictly differentiate between post-stroke pneumonia (22) and ventilator-associated pneumonia (26).

Some investigators have raised concerns that early extubation after the thrombectomy may induce blood pressure fluctuations and precipitate hyperperfusion syndrome (27, 28), particularly in the context of prolonged onset-to-recanalization time and the inherently high risk of hemorrhagic transformation in our study population. However, no association was found between intubation duration and hemorrhagic transformation in the present study. Of note, prolonged intubation duration in some patients may itself be a consequence of hemorrhagic transformation (11), and the causal relationship between these two factors warrants further investigation.

Extubation failure leading to reintubation is an important factor associated with poor outcome (12, 29, 30). In our study, only three patients (2.3%) required reintubation: one due to laryngeal edema and two due to severe hypoxemia secondary to pulmonary infection. This reintubation rate is much lower than that reported in the literature (12, 30), possibly because all patients underwent a spontaneous breathing trial (SBT) before extubation and due to the relatively conservative extubation strategy adopted in our institution. As emphasized by the ENESTROKE study (9), one should not extubate early for the mere sake of early extubation; premature extubation that ignores the patient‘s underlying disease severity may be unreasonable.

Several limitations of this study should be considered when interpreting the findings. First, the retrospective design precludes establishing causality. Although multivariable analysis adjusted for several key confounders, unmeasured factors may have influenced the results (31, 32). Second, we excluded 25 patients who could not be extubated within 96 h post-thrombectomy and those requiring mechanical ventilation >96 h. These excluded patients had more severe disease and higher mortality, suggesting that our findings may underestimate the strength of the association between delayed extubation and poor outcomes. Third, as a single center study with a limited sample size, the generalizability of our findings requires validation in larger, multicenter studies. Fourth, the diagnosis of pneumonia relied on clinical records and may be subject to bias; moreover, we did not strictly differentiate between ventilator-associated pneumonia and post-stroke pneumonia. Additionally, the retrospective design precluded accurate determination of the temporal sequence between pneumonia and delayed extubation, making it difficult to rule out reverse causality. Nonetheless, the consistent association after adjusting for major confounders suggests that intubation duration may serve as a useful prognostic indicator in this patient population.

## 5 Conclusion

Prolonged intubation duration after thrombectomy under GA for AIS is associated with poor outcome and pneumonia. These findings may help identify patients at higher risk of poor outcomes, but require further validation in prospective studies.

## Data Availability

The raw data supporting the conclusions of this article will be made available by the authors, without undue reservation.
