# Supplementary material for: Intubation duration after general anesthesia for thrombectomy in acute ischemic stroke: a cohort study
Source: Front Neurol. 2026 Aug 27;17:1937683. doi: 10.3389/fneur.2026.1937683 (PMC13560773; doi:10.3389/fneur.2026.1937683)
Supplement: Supplementary file 1 [file Supplementary_file_1.docx]

**Supplementary materials content**

Supplementary Table S1. Baseline characteristics between conscious sedation and general anesthesia.

Supplementary Table S2. Baseline characteristics between intubation duration >96 hours or discharge with intubation and intubation duration ≤ 96 hours.

Supplementary Table S3. Sensitivity analysis: association between intubation duration and poor functional outcome.

Supplementary Figure S1. ROC curves of intubation duration for predicating outcomes.

Supplementary Table S1. Baseline characteristics between conscious sedation and general anesthesia.

|  | Conscious sedation  (N=190) | General anesthesia  (N=158) | P value |
| --- | --- | --- | --- |
| Male, n (%) | 120 (63.2%) | 102 (64.6%) | 0.78 |
| Age, mean (SD) | 62.9 (15.4) | 63.4 (12.3) | 0.01 |
| Medical History, n (%) |  |  |  |
| Hypertension | 119 (62.6%) | 108 (68.4%) | 0.26 |
| Diabetes | 52 (27.4%) | 48 (30.4%) | 0.54 |
| Atrial fibrillation | 54 (28.4%) | 31 (19.6%) | 0.06 |
| Stroke | 32 (16.8%) | 23 (14.6%) | 0.56 |
| Coronary heart disease | 54 (28.4%) | 28 (17.7%) | 0.02 |
| Smoking | 57 (30.0%) | 62 (39.2%) | 0.07 |
| Baseline NIHSS, median (IQR) | 13 (9-18) | 13 (8-18) | 0.59 |
| Baseline GCS, median (IQR) | 12 (10-14) | 12 (10-14) | 0.87 |
| Baseline ASPECTS, median (IQR) | 6 (5-8) | 6 (5-8) | 0.73 |
| Baseline SBP, mmHg, mean (SD) | 147.2 (24.1) | 149.0 (22.3) | 0.27 |
| Baseline DBP, mmHg, mean (SD) | 85.4 (14.5) | 85.7 (14.5) | 0.66 |
| Cardiogenic embolism, n (%) | 54 (28.4%) | 36 (22.8%) | 0.47 |
| Decreased or absent cough reflex, n (%) | 113 (59.5%) | 92 (58.2%) | 0.81 |
| Baseline laboratory test, mean (SD) |  |  |  |
| estimated glomerular filtration rate, mL/min per 1.73 m^2^ | 100.8 (32.3) | 97.3 (22.0) | 0.002 |
| Blood glucose, mmol/l | 8.4 (4.6) | 7.4 (2.9) | <0.001 |
| White blood count, 10^9^/L | 9.4 (3.4) | 9.3 (3.5) | 0.39 |
| Vertebrobasilar artery occlusion, n (%) | 34 (17.9%) | 47 (29.8%) | 0.01 |
| Intravenous thrombolysis, n (%) | 45 (23.7%) | 37 (23.4%) | 0.95 |
| ASTIN/SIR scale 3-4, n (%) | 32 (16.9) | 32 (20.3) | 0.25 |
| Door to puncture time, min, median (IQR) | 78 (65-91) | 108 (70-162) | <0.001 |
| Puncture to recanalization time, min, median (IQR) | 65 (50-80) | 105 (73–135) | <0.001 |
| Onset to recanalization time, min, median (IQR) | 565 (420-797) | 607 (428-850) | 0.34 |
| mTICI scale 2b-3, n (%) | 168 (88.4) | 143 (91.1) | 0.62 |
| 90-d mRS 3-6, n (%) | 124 (65.3) | 94 (59.5) | 0.26 |
| 90-d mRS, n (%) |  |  | 0.37 |
| 0 | 24 (12.6%) | 20 (12.7%) |  |
| 1 | 13 (6.8%) | 23 (14.6%) |  |
| 2 | 29 (15.3%) | 21 (13.3%) |  |
| 3 | 26 (13.7%) | 18 (11.4%) |  |
| 4 | 42 (22.1%) | 28 (17.7%) |  |
| 5 | 16 (8.4%) | 15 (9.5%) |  |
| 6 | 40 (21.1%) | 33 (20.9%) |  |
| Hemorrhagic transformation, n (%) |  |  | 0.86 |
| Hemorrhagic infarction 1 | 13 (6.8%) | 9 (5.7%) |  |
| Hemorrhagic infarction 2 | 12 (6.3%) | 11 (7.0%) |  |
| Parenchymal hemorrhage 1 | 9 (4.7%) | 9 (5.7%) |  |
| Parenchymal hemorrhage 2 | 14 (7.4%) | 16 (10.1%) |  |
| Pneumonia, n (%) | 84 (44.2%) | 90 (57.0%) | 0.02 |
| 90-d mortality | 40 (21.1%) | 33 (20.9%) | 0.97 |

SD, standard deviation; NIHSS, National Institutes of Health Stroke Scale score; IQR, interquartile ranges; GCS, Glasgow Coma Scale; ASPECTS, Alberta Stroke Program Early CT Score; SBP, systolic blood pressure; DBP, diastolic blood pressure; ASTIN/SIR, American Society of Interventional and Therapeutic Neuroradiology/Society of Interventional Radiology; mTICI, modified Thrombolysis in Cerebral Infarction; mRS, modified Rankin Scale.

Supplementary Table S2. Baseline characteristics between intubation duration >96 hours or discharge with intubation and intubation duration ≤ 96 hours.

|  | Intubation duration > 96 hours or discharge with intubation (N=25) | Intubation duration≤ 96 hours  (N=133) | P value |
| --- | --- | --- | --- |
| Male, n (%) | 11 (44.0) | 91 (68.4) | 0.02 |
| Age, mean (SD) | 65.5 (14.2) | 63.1 (11.9) | 0.36 |
| Medical History, n (%) |  |  |  |
| Hypertension | 13 (52.0) | 95 (71.4) | 0.05 |
| Diabetes | 6 (24.0) | 42 (31.6) | 0.45 |
| Atrial fibrillation | 7 (28.0) | 24 (18.0) | 0.25 |
| Stroke | 3 (12.0) | 20 (15.0) | 0.69 |
| Coronary heart disease | 8 (32.0) | 20 (15.0) | 0.04 |
| Smoking | 7 (28.0) | 55 (41.4) | 0.21 |
| Baseline NIHSS, median (IQR) | 16 (13-23) | 12 (8-17) | 0.004 |
| Baseline GCS, median (IQR) | 10 (7-13) | 13 (11-14) | 0.01 |
| Baseline ASPECTS, median (IQR) | 5 (4-7) | 6 (5-8) | 0.006 |
| Baseline SBP, mmHg, mean (SD) | 142.9 (27.9) | 150.2 (21.0) | 0.14 |
| Baseline DBP, mmHg, mean (SD) | 83.0 (17.3) | 86.2 (14.0) | 0.23 |
| Cardiogenic embolism, n (%) | 8 (32.0) | 28 (21.1) | 0.23 |
| Decreased or absent cough reflex, n (%) | 19 (76.0%) | 73 (54.9%) | 0.05 |
| Baseline laboratory test, mean (SD) |  |  |  |
| estimated glomerular filtration rate, mL/min per 1.73 m^2^ | 90.5 (22.6) | 98.5 (21.7) | 0.09 |
| Blood glucose, mmol/l | 8.3 (2.1) | 7.2 (3.1) | 0.28 |
| White blood count, 10^9^/L | 9.5 (3.6) | 9.3 (3.6) | 0.71 |
| Vertebrobasilar artery occlusion, n (%) | 6 (24.0) | 41 (30.8) | 0.08 |
| Intravenous thrombolysis, n (%) | 6 (24.0) | 31 (23.3) | 0.94 |
| ASITN/SIR scale 3-4, median (IQR) | 3 (12.0) | 29 (21.8) | 0.008 |
| Onset to recanalization time, min, median (IQR) | 585 (377-750) | 613 (430-855) | 0.84 |
| mTICI scale 2b-3, n (%) | 21 (84.0) | 123 (92.4) | 0.05 |
| Off-hours, n (%) | 9 (36.0) | 41 (30.8) | 0.61 |
| 90-d mRS 3-6, n (%) | 25 (100.0) | 64 (48.1) | <0.001 |
| 90-d mRS, n (%) |  |  | <0.001 |
| 0 | 0 (0) | 20 (15.0) |  |
| 1 | 0 (0) | 23 (17.3) |  |
| 2 | 0 (0) | 21 (15.8) |  |
| 3 | 1 (4.0) | 17 (12.8) |  |
| 4 | 3 (12.0) | 25 (18.8) |  |
| 5 | 2 (8.0) | 13 (9.8) |  |
| 6 | 19 (76.0) | 14 (10.5) |  |
| Hemorrhagic transformation, n (%) |  |  | <0.001 |
| Hemorrhagic infarction 1 | 0 (0) | 9 (6.8) |  |
| Hemorrhagic infarction 2 | 3 (12.0) | 8 (6.0) |  |
| Parenchymal hemorrhage 1 | 3 (12.0) | 6 (4.5) |  |
| Parenchymal hemorrhage 2 | 8 (32.0) | 8 (6.0) |  |
| Pneumonia, n (%) | 16 (64.0) | 74 (55.6) | 0.44 |
| 90-d mortality, n (%) | 19 (76.0) | 14 (10.5) | <0.001 |

SD, standard deviation; NIHSS, National Institutes of Health Stroke Scale score; IQR, interquartile ranges; GCS, Glasgow Coma Scale; ASPECTS, Alberta Stroke Program Early CT Score; SBP, systolic blood pressure; DBP, diastolic blood pressure; ASITN/SIR, American Society of Interventional and Therapeutic Neuroradiology/Society of Interventional Radiology; mTICI, modified Thrombolysis in Cerebral Infarction; mRS, modified Rankin Scale.

Supplementary Table S3. Sensitivity analysis: association between intubation duration and poor functional outcome.

|  | 90-d mRS 3-6 | | Pneumonia | |
| --- | --- | --- | --- | --- |
|  | aOR (95% CI) | P value | aOR (95% CI) | P value |
| 24-48h vs ≤24h | 1.32 (0.50-3.49) | 0.57 | 1.08 (0.39-2.96) | 0.87 |
| >48h vs ≤24h | 2.81 (1.15-6.87) | 0.02 | 3.10 (1.09-8.80) | 0.03 |

aOR, adjusted odd ratio; CI, confidence interval; mRS, modified Rankin Scale.

Adjusting for age, sex, baseline National Institutes of Health Stroke Scale score, baseline Glasgow Coma Scale, baseline Alberta Stroke Program Early CT Score, cough reflex, baseline estimated glomerular filtration rate, intravenous thrombolysis and atrial fibrillation.

Supplementary Figure S1. ROC curves of intubation duration for predicating outcomes. A. Poor outcome at 90 days, AUC=0.71 (95% CI 0.62-0.79); B. Post-stroke pneumonia, AUC=0.72 (95% CI 0.63-0.81). ROC, receiver operating characteristic; AUC, area under curves, CI, confidential interval.
